# Largescale Transcriptomics Analysis Suggests Over-Expression of BGH3, MMP9 and PDIA3 in Oral Squamous Cell Carcinoma

**DOI:** 10.1371/journal.pone.0146530

**Published:** 2016-01-08

**Authors:** Yuan He, Fangyang Shao, Weidong Pi, Cong Shi, Yujia Chen, Diping Gong, Bingjie Wang, Zhiwei Cao, Kailin Tang

**Affiliations:** 1 Department of Oral Medicine, School of Stomatology, Tongji University, Shanghai, 200092, China; 2 School of Life Science and Technology, Tongji University, Shanghai, 200092, China; 3 Advanced Institute of Translational Medicine, Tongji University, Shanghai, 200092, China; University of the Sunshine Coast, AUSTRALIA

## Abstract

Oral squamous cell carcinoma (OSCC) has been reported as the most prevalent cancer of the head and neck region, while early diagnosis remains challenging. Here we took a comprehensive bioinformatics study on microarray data of 326 OSCC clinical samples with control of 165 normal tissues. The cell interaction pathways of ECM-receptor interaction and focal adhesion were found to be significantly regulated in OSCC samples. Further analysis of the topological properties and expression consistency identified that three hub genes in the gene interaction network, MMP9, PDIA3 and BGH3, were consistently up-expressed in OSCC samples. When being validated on additional microarray datasets of 41 OSCC samples, the validation rate of over-expressed BGH3, MMP9, and PDIA3 reached 90%, 90% and 84% respectively. At last, immuno-histochemical assays were done to test the protein expression of the three genes on newly collected clinical samples of 35 OSCC, 20 samples of pre-OSCC stage, and 12 normal oral mucosa specimens. Their protein expression levels were also found to progressively increase from normal mucosa to pre-OSCC stage and further to OSCC (ANOVA p = 0.000), suggesting their key roles in OSCC pathogenesis. Based on above solid validation, we propose BGH3, MMP9 and PDIA3 might be further explored as potential biomarkers to aid OSCC diagnosis.

## Introduction

As the most prevalent cancer of the head and neck region, oral squamous cell carcinoma (OSCC) accounts for 3–4% of all cancer cases[1]. Every year, an estimated 3 million new cases occur worldwide, and the overall 5-year survival rate for OSCC is only 50%[2]. The use of microarray technology to investigate OSCC pathogenesis has been widely used in recent years and the fast accumulation of microarray data has provided opportunities to investigate the mechanism of OSCC disease. More importantly, several articles have focused on exploring the differentially expressed genes (DEGs) as potential biomarkers for OSCC [3,4,5]. For instance, Koh-Ichi Nakashiro et.al. studied gene profiles in 10 primary OSCCs and 10 human OSCC cell lines using Applied Biosystems Human Genome Survey Arrays. They identified Akt1 as the only gene that was expressed in OSCC tissues and cultured cells, but not in non-neoplastic tissues and cells[6]. Kim Yong-Deok et.al. investigated the gene expression of tumor-normal paired tissues from five OSCC patients. After validated by qRT-PCR, four genes (ADAM15, CDC7, IL12RB2 and TNFRSF8) have been proposed as potential biomarkers of OSCC[7]. Chu Chen et. al. identified differential expressed genes using a training set of 119 OSCC patients and 35 controls then validated the selected genes in an internal testing set of 48 invasive OSCC and 10 controls and further on an external testing set of 42 head and neck squamous cell carcinoma cases and 14 controls[8]. Although insightful, it is clearly noticed that a large discrepancy exists cross different studies at mRNA level as well as protein level[9]. The potential reasons that may cause diverse even contradicting conclusions between different studies often include different sample size, different experimental platforms, and even different statistical methods[10]. Thus, deriving DEGs from sample sets as large as possible, and solid validation on independent clinical samples at not only mRNA level but also protein expression level would be more significant when potential biomarkers are explored.

In this study, a comprehensive bioinformatics analysis was performed on the largest dataset of 326 OSCC samples with control of 165 normal tissues with different experimental platforms to identify critical genes related to OSCC pathogenesis. Then solid validation on totally independent clinical samples was carried at both mRNA level (41 OSCC samples) and protein level (35 OSCC samples, 20 pre-OSCC stage samples, and 12 normal oral mucosa specimens) by immune-histochemical (IHC) assay. Our results found consistent overexpression of BGH3, MMP9 and PDIA3 in OSCC samples.

## Materials and Methods

### Acquisition of microarray data

The data were downloaded from the GEO database (http://www.ncbi.nlm.nih.gov/geo/) and were selected based on the following criteria to ensure the reliability of the data analyses: (1) availability of raw microarray data; (2) inclusion of both oral squamous cell carcinoma and normal control (either adjacent normal or oral mucosa from healthy individuals); and (3) more than 10 tumor samples. Consequently, there were 6 datasets [11,12,13,14,15,16] using Affymetrix microarray that met our requirements (S1 Table). A total of 481 samples (326 OSCC and 165 normal controls) were included in this analysis. To ensure abundant availability of information for subsequent analyses, GSE9844/GSE30784/GSE31056 were labeled as “group I” (which was from Affymetrix Human Genome U133A Plus 2.0 array) and the remaining datasets were labeled as “group II” (which consisted of the former version of Affymetrix such as Human Genome U133A, Human Genome U95Av2 and Human Genome U133A2.0) according to their microarray platform.

### Identification of differential expressed genes

Array data files with a.CEL extension were normalized using the GeneChip Robust Multi-array Analysis algorithm implemented by the R package (http://www.bioconductor.org/). The probe sets with probe identification (ID) marked as AFFX internal control were excluded.

The RankProd method[17], which ranks genes based on their differential expression between pairs of samples within studies, was utilized to avoid batch and platform-related effects (they usually occur when directly combining expression values from different studies). Specifically, fold change was used to compare and rank the genes within each dataset. These ranks were then aggregated to produce an overall score for the genes across all datasets, forming a ranked gene list.

DEGs were selected with a false discovery rate (FDR) less than 0.01 and fold-change ≥2 in I or II groups.

### PPI network construction and clustering

The 4 public databases that contain protein-protein interaction information for human proteins, namely, the Human Protein Reference Database (HPRD)[18], Molecular Interaction database (MINT)[19], Intact[20] and Database of Interacting Proteins (DIP)[21], were integrated in our study. The protein interaction pairs extracted from these databases were integrated to form a human background network. DEGs were then mapped to this background network. The network was simplified to a minimum network containing the derived proteins through the Steiner minimal tree algorithm[22].

The densely connected and possibly overlapping regions within the Cytoscape network were determined by ClusterONE (Clustering with Overlapping Neighborhood Expansion)[23], which detects densely connected regions by essentially looking for groups of nodes in a network with high cohesiveness. Densely connected regions are those for which the sum of the edges between two nodes in one selected group are significantly larger than the sum of the edges at the location where one endpoint lies within the selected group and the other lies outside the selected group. The minimum size (at least 7 nodes in a sub-network) and the minimum density (at least 0.3 in a sub-network) are defined to decompose the network. It provided an efficient way to assess how well a given subgraph fits the two structural properties: it should contain many reliable interactions between its subunits, and it should be well-separated from the rest of the network.

Given the topology of the PPI (protein-protein interaction) network, the distance from each gene to DEGs was defined as the sum of the shortest distance between the two nodes in a subnet. This parameter can be used to measure the influence of each gene. The nodes in the subnet whose average shortest distance to DEGs was less than the lower quartile in the subnet and the value of degree was more than 6 were identified as the hub genes [24].

### Immunohistochemical assay

Patients diagnosed with OSCC or oral leukoplakia (OLK, pre-OSCC stage) were enrolled at the Department of Oral and Maxillofacial Surgery, School of Stomatology, Tongji University from 2008 to 2014. Normal oral mucosa (NOM) samples were obtained during teeth extraction, gingivectomy and other minor surgical operations. All subjects who participated in this study provided written consent, and the project was approved by the Research Ethics Committee of Tongji University.

All surgically resected specimens were formalin-fixed and paraffin-embedded using conventional techniques. Histopathological evaluation was performed according to the criteria of the international collaborative group on oral white lesions and the World Health Organization on oral cancers by two experienced histopathologists in a double-blind manner.

Rabbit monoclonal antibodies against BGH3 (ab170874), PDIA3 (ab154191) and MMP-9 (ab76003) were purchased from Abcam Inc. (Boston, USA). Formalin-fixed, paraffin-embedded tumor sections (4–5 μm) were prepared, dried for 1 h at 60°C, dewaxed in xylene, and rehydrated with a gradation of ethanol solutions ending with distilled water. Heat-mediated antigen retrieval was performed in citrate buffer (pH 6.0) using a microwave oven according to the instructions provided with the antibody. After being allowed to cool naturally, 3–4% H_2_O_2_ was used to quench endogenous peroxidase activity. Next, the sections were incubated with primary antibodies (diluted with PBS at 1:100) for 1h at room-temperature. Subsequently, the sections were incubated with the corresponding secondary antibody (anti-rabbit IgG, HRP-linked antibody #7074, Cell Signaling Technology, Shanghai, China) for 30 min at room temperature. The antibody reaction was visualized using diaminobenzidine (DAB) chromogen and counterstained with hematoxylin. Primary antibody was omitted for sections used as negative controls.

For semi-quantitative evaluation of IHC staining, an immunoreactivity score (IRS) system was applied as previously described[25]. Cells of three random fields in each section were visualized at 400× magnification and counted by two skilled technicians. The staining intensity (1, weak; 2, moderate; 3, strong) and positive cell percentage were multiplied to calculate the IHC score. Statistical analyses were performed with SPSS (Statistical Package for the Social Sciences) software version 20.0 (SPSS Inc., Chicago, IL). The independent sample t-test was used for simple comparisons between two groups. ANOVA was used to compare data between more than two groups. The Pearson correlation test was performed to determine the relationship between the expression levels of two proteins, and the chi-square test was used to establish the statistical significance of the expression rate[26].

## Results

### DEGs significantly enriched in the cell interaction pathways

Different data set will provide different perspectives. Combining multiple data sets for an integrative analysis would not only expand sample size but also obtain better statistical power for more consistent conclusions. In this study, we collected 326 OSCC and 165 normal controls and divided them in two groups (I and II) according to their array platform. There were 216 OSCC samples and 130 controls in group I (Affymetrix Human Genome U133A Plus 2.0) and 110 OSCC samples and 35 controls in group II (Affymetrix such as Human Genome U133A, Human Genome U95Av2 and Human Genome U133A2.0).

DEGs were selected in I and II groups with both fold change above two and false discovery rate (FDR) less than 0.05. A total of 1046 and 246 DEGs in group I and group II, respectively, were screened. The 205 overlapping DEGs between the two groups were used for the subsequent analyses, and among them, 113 genes were up-regulated and 92 genes were down-regulated (S1 File).

The top enriched pathways often indicate the most prominent functions of the DEGs. DAVID (Database for Annotation, Visualization and Integrated Discovery) was used to select significantly enriched KEGG (Kyoto Encyclopedia of Genes and Genomes) pathways[27]. In addition to those extensively studied signaling pathways repeatedly documented to be involved in OSCC, such as cell proliferation, cell cycle and apoptosis, our work showed that the ECM-receptor interaction and focal adhesion pathways are significantly enriched in OSCC samples (Table 1). It is noted that the two pathways are actually closely interacting with many overlapping genes such as collagens, integrins and laminins etc., according to KEGG annotation. In Table 1, those genes in ECM-receptor interaction pathway are fully covered by focal adhesion, suggesting the high importance of cell interaction in OSCC disease.

**Table 1:** The enriched pathways regulated by DEGs in OSCC.

Enriched Terms	FDR	DEGs participated in each pathway
ECM-receptor interaction	1.57E-08	COL4A6, LAMB3, LAMC2, SPP1, COL6A3, COL4A2, COL11A1, COL1A1, COL4A1, LAMB1, ITGA6, COL5A2, COL3A1, ITGAV, TNC, COL1A2
Focal adhesion	5.54E-04	COL4A6, LAMB3, LAMC2, SPP1, COL6A3, COL4A2, COL11A1, COL1A1, COL4A1, LAMB1, ITGA6, COL5A2, COL3A1, ITGAV, TNC, COL1A2, ACTN1

### Hub genes identified in PPI network

Genes with significant interaction partners, also called hubs, are involved in many cellular processes. Many hubs in differential interaction network often serve as key components of cancer-associated pathways and would be used to discover cancer-induced genes[28]. Therefore, studying interaction networks is useful for deciphering the molecular basis of diseases. As DEGs are often downstream effects of gene network disturbing, those hub genes connecting the most DEGs may be more important in initiating the gene regulating network of a disease state. That is to say, the common neighbors of many DEGs might be important up-stream initiators or even disease driver genes. The above identified DEGs were then mapped to the integrated protein-protein interaction (PPI) data for a network. For better clarification, the network was simplified to a minimum network containing the derived proteins through the Steiner minimal tree algorithm[22], in which the DEGs could be linked together through direct interaction or through one intermediary at the PPI level.

**Fig 1:**
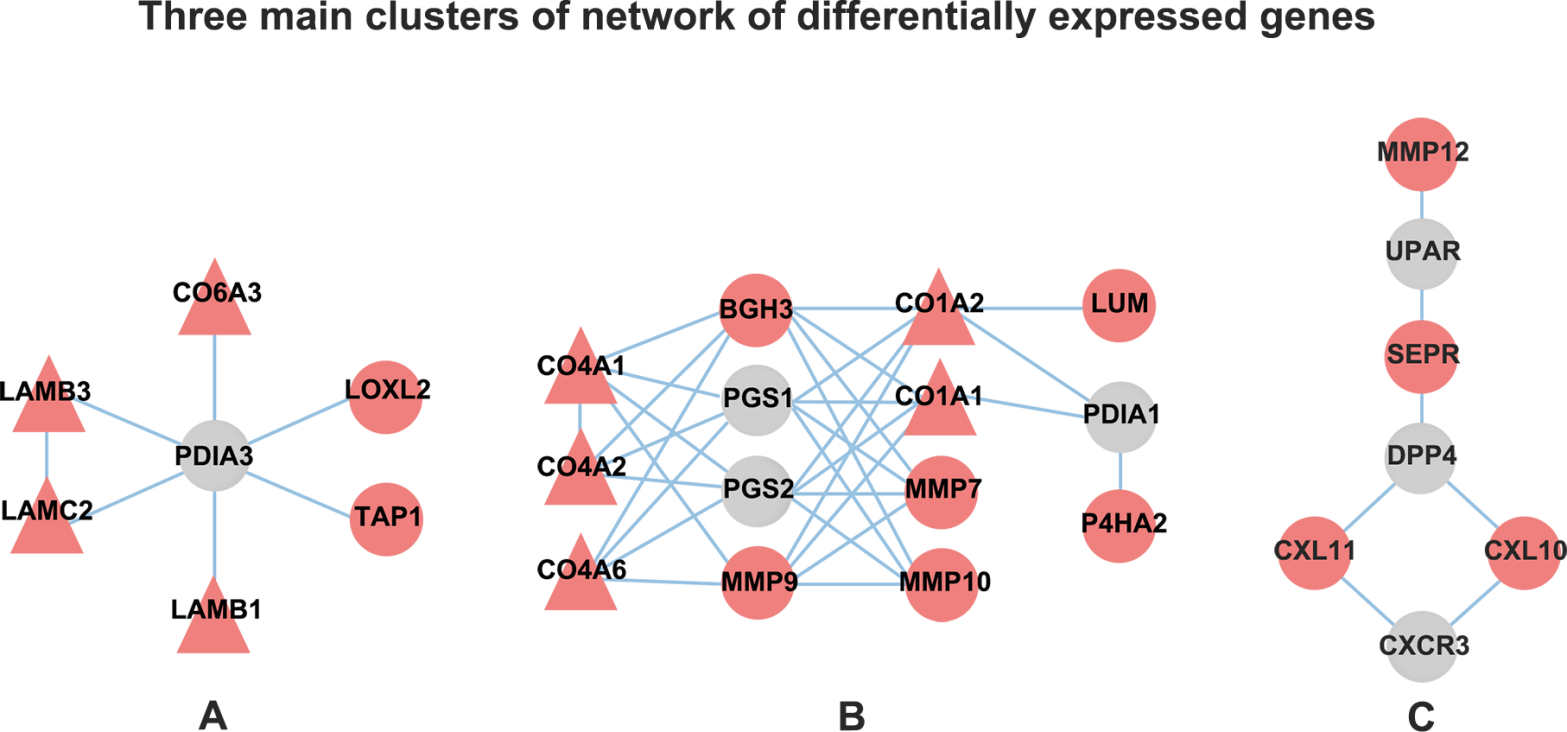
The three main dense modules of DEGs. Each circled or triangle point represents a node in the network and each line represents the interaction between two nodes. Red node: up-expressed gene; green node: down-expressed gene; gray node: background gene; triangle node: gene included in the enriched pathway.

Genes in networks always have the structures in which genes are more closely connected. This kind of sub-network is termed as a network module or cluster. Densely connected regions in the network was detected by essentially looking for groups of nodes with high cohesiveness, which provided an efficient way to assess how well a given subgraph fits the two structural properties: it should contain many reliable interactions between its subunits, and it should be well-separated from the rest of the network. After clustering, three primary densely connected modules were obtained, whose size was at least 7 nodes, the density was above 0.3 and p-value was less than 0.05 (Table A in S2 File). There were 7 nodes in module A and module C, while 14 nodes in module B (Fig 1). Module A and module B were mainly related to the focal adhesion and the ECM-receptor interaction. While module C were more involved into the chemokine-receptor interactions.

In module A, PDIA3 situates in the core of this subnet which interacted with all other DEGs. Although PDIA3 was not screened as a DEG in the above analysis, it is mildly but consistently up-regulated with 1.3-fold in group A and 1.8-fold in group B with both FDR less than 0.01. Furthermore, it is surrounded by cluster of DEGs indicating its potential importance. In module B, the highest dense cluster, four genes (MMP9, BGH3, PGS1 and PGS2) were identified as the hub genes according to the less average shorst distances to DEGs (1.36, 1.27, 1.45, 1.45) and the higher degrees(6, 7, 7, 7) (Table B in S2 File), implying that these four genes have high connectivity and are important in the subnet. It is also noticed, despite the fact that PGS1 and PGS2 were not filtered as DEGs in above section, the close relationship between PGS1, PGS2 and the DEGs inclined their special place in OSCC. There is no distinct hub genes in module C.

### Positive Validation at mRNA level

We calculated the validated rate of the above hub genes at mRNA level via training data and independent dataset. The expression of these genes in the original training datasets were firstly checked. Their fold change (FC) and up/down expression were summarized into Table 2. To test the generalizability of the hub genes, two other independent microarray datasets were downloaded from GEO. One contains 24 paired tumor and normal samples from GSE42743[29] with platform of Affymetrix U133 plus 2.0 array. The other covers 27 OSCC patients compared with 4 independent controls and 1 pooled control from health from GSE23558[30] with Agilent-014850 Whole Human Genome array.

**Table 2:** Fold change and validated rate^*^ of hub genes in training data and independent testing data.

Gene	Group I in training data				Group II in training data				Total in training data	Independent testing data 1		Independent testing data 2		Total in testing data
	FC	GSE9844	GSE30784	GSE31056	FC	GSE13601	GSE3524	GSE23036		FC	GSE42743	FC	GSE23558	
MMP9	6.44	92.3%	98.2%	100%	2.74	100%	75%	100%	98.4%	5.13	90%	2.92	90%	90%
BGH3	4.25	92.3%	96.4%	100%	4.11	100%	100%	90.5%	95.6%	2.47	88%	2.88	92%	90%
PDIA3	1.3	80.8%	76.6%	91.3%	1.88	93.5%	100%	84.1%	81.9%	1.52	86%	1.56	82%	84%
PGS1	1.82	30.8%	87.4%	87.0%	1.23	80.6%	68.8%	61.9%	77.8%	1.96	82%	1.53(p = 0.054)	88%	85%
PGS2	0.57	80.8%	64.1%	87.0%	NA	NA	43.80%	73.0%	71.2%	0.53	71%	0.15	100%	86%

* The validated rate was the proportion of the OSCC samples in each training dataset and testing data, in which the intensity of the hub gene was larger than the average level of that in control samples. (To PGS2, it was calculated as the proportion of OSCC samples whose intersity was lower than the gene’s average value in control samples.)

MMP9, BGH3, PDIA3, PGS1 were up-expressed, while PGS2 was down-expressed in OSCC samples. It can be seen that the fold change of MMP9 and BGH3 were above two in all conditions and the two genes had a consistent up-expression in most OSCC samples. The up-expressed FC of PDIA3 ranged from 1.3 fold to 1.88 fold. To PGS1, the up-expressed FC was from 1.23 to 1.96. While to PGS2, the down-expressed FC was from 0.15 to 0.57. The expression of BGH3, MMP9, and PDIA3 in testing data was up-regulated in 90%, 90%, and 84% of samples, respectively.

In the training and testing data, the candidate genes PDIA3, MMP9 and BGH3 were over-expressed in most OSCC samples, while the expression of the candidate genes PGS1 and PGS2 exhibited greater fluctuation between samples than PDIA3, MMP9 and BGH3. Considering the overall validated rate in total training and testing data, we selected BGH3, MMP9 and PDIA3 for further validation on protein level.

### Up-Expression of hub genes at protein level

**Fig 2:**
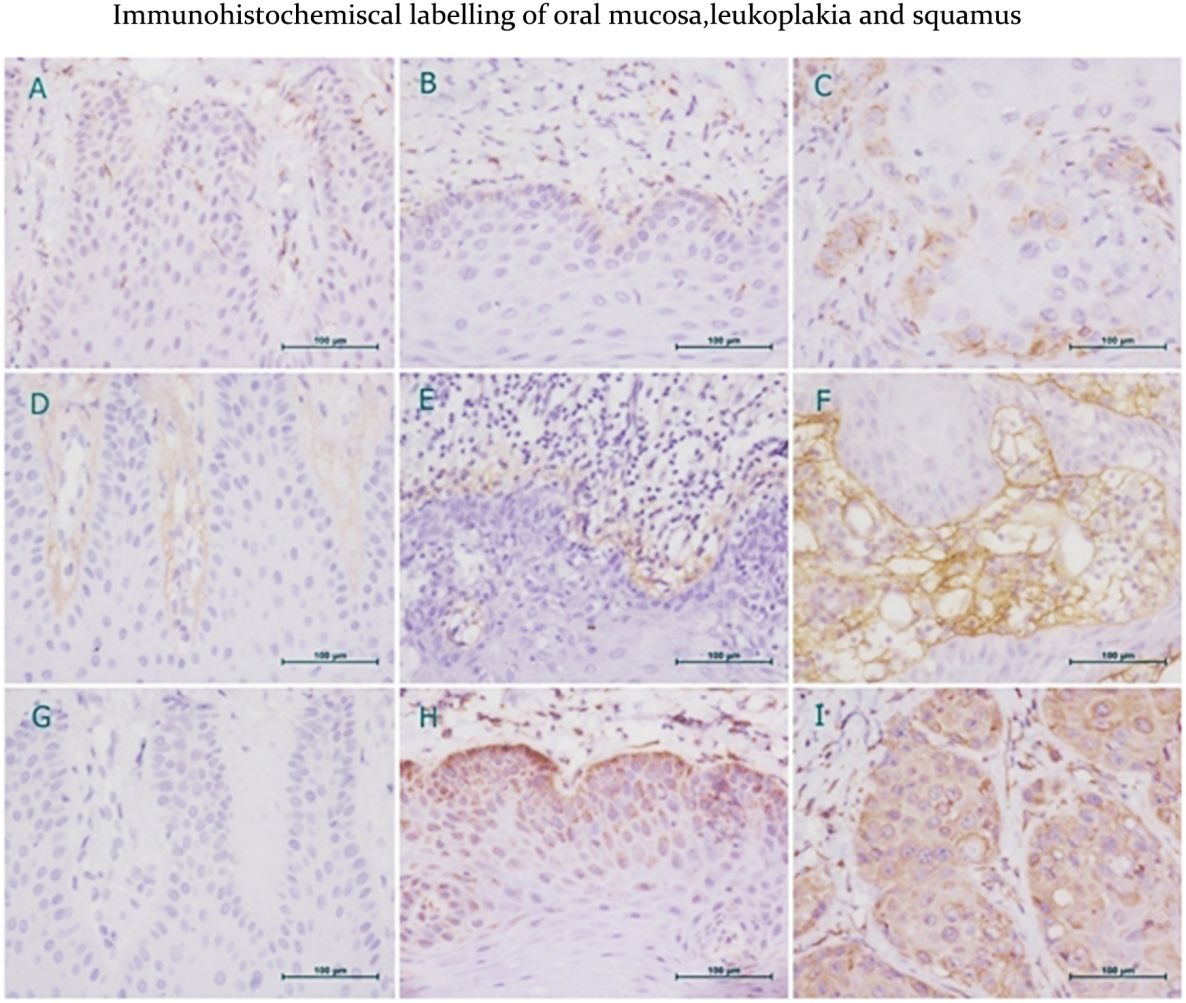
Immunohistochemical labeling of normal oral mucosa (A,D,G), oral leukoplakia (B,E,H) and oral squamous cell carcinoma (G,H,I) for MMP9 (A,B,C), BGH3 (D,E,F) and PDIA3 (G,H,I). Brown staining represents positive expression of proteins. MMP9 expression in stroma cells is low (A). Positive MMP9 cytoplasmic staining of basal cells in OLK (B). Strong nuclear and cytoplasmic MMP9 staining of OSCC (C). Negative BGH3 expression in NOM (D). Weak or moderate BGH3 staining of the basement membrane in OLK (E). Strong positive BGH3 expression in tumor basement membrane and extracellular skeletal stroma of OSCC (F). Negative PDIA3 staining in NOM (G). Moderate to strong PDIA3 staining in the cytoplasm and nucleus of OLK epithelium cells (H). High PDIA3 expression in the cytoplasm and cell membrane of OSCC (I).

To further validate the up-expression of the gene at protein level, we collected new clinical samples of 35 OSCC, 20 OLK (pre-OSCC stage), and 12 NOM specimens. The clinical parameters of the patients and the protein-positive expression rates for each group was listed in S2 Table. **Immunohistochemical labeling** was done to check the protein expression of the 3 genes, as Fig 2 indicates. It can be seen that BGH3 is mainly located in the basement membrane (BM) and intercellular skeletal substance, MMP9 is located in the cytoplasm and nucleus of stromal cells, while PDIA3 is mainly located in the cytoplasm and membrane (Fig 2).

The IHC scores of the three proteins expressed in each group are shown in Table 3. It can be seen that, the IHC scores of MMP9, BGH3 and PDIA3 progressively increased from 21.2, 12.5 and 0.0 in NOM to 38.9, 42.5 and 99.4 in OLK(pre-OSCC stage), and further to 116.8, 99.9 and 215.0 in OSCC (ANOVA p = 0.000), suggesting their key roles in OSCC pathogenesis development. In addition, the correlation analysis showed a moderate positive linear association between MMP9 and BGH3 expression in OSCC and OLK samples.

**Table 3:** Means and standard deviation of IHC scores, correlation between proteins in each group.

Group	IHC mean(SD)			Pearson Correlation(p-value/R-value)		
	MMP9	BGH3	PDIA3	MMP9/BGH3	BGH3/PDIA3	PDIA3/MMP9
NOM	21.2(16.0)	12.5(14.4)	0.0	0.285/0.321	▲	▲
OLK	38.9(55.3)	42.5(48.2)	99.4(108.5)	0.001/0.685☆	0.476/0.169	0.227/0.283
OSCC	116.8(65.2)	99.9(72.3)	215.0(77.7)	0.002/0.512☆	0.240/0.204	0.762/0.053
ANOVA test	P = 0.000★	P = 0.000★	P = 0.000★			

★IHC score mean difference is significant at the 0.001 level.

☆correlation is significant at 0.01level(2-tailed).

▲can’t be calculated.

## Discussions

Over the past decades, many cancer-related studies have focused on genes involved in transcription regulation and cell cycle control. In this study, a transcriptomics analysis was performed on a large scale microarray dataset from GEO to study not only DEGs but also hub genes among DEGs. The focal adhesion and ECM-receptor interaction pathways were found to be significantly regulated in OSCC samples. Consistent overexpression of BGH3, MMP9 and PDIA3 in OSCC samples were confirmed at both mRNA level and protein level by immunohistochemical(IHC) assay.

The maximally altered pathway was the extracellular matrix (ECM)-receptor interaction pathway, consisting of a complex mixture of structural and functional macromolecules. The biological functions of ECM proteins have been underestimated for a long time[31]. These molecules serve an important role in tissue and organ morphogenesis and in the maintenance of cellular and tissue structure and function. Interactions between cells and the ECM led to the direct or indirect control of cellular processes of adhesion, migration, differentiation, proliferation, and apoptosis[32].

The other enriched pathway was focal adhesion pathway. Focal adhesions are the sub-cellular structures that mediate the regulatory effects (i.e., signaling events) of a cell in response to ECM adhesion[33]. Cell-matrix adhesions play essential roles in important biological processes including cell motility, cell proliferation, cell differentiation, regulation of gene expression and cell survival. Recently, the microenvironment has gained increasing attention in regards to many biological processes[34]. The balance between cell adhesion and extracellular molecules is important for normal cell survival[35]. Cancers are currently regarded as heterogeneous multi-cellular entities that contain cells of multiple lineages that interact with the extracellular matrix (ECM). These interactions favor cancer cell proliferation, movement, differentiation, and ECM metabolism and simultaneously restrict cell death, stationary polarized growth, and ECM stability[36]. Overall, the cell interaction pathways of focal adhesion and ECM-receptor interaction were significantly regulated in OSCC samples.

After bioinformatics analysis and solid validation on independent clinical samples at mRNA and protein level, BGH3, MMP9 and PDIA3 have been identified as three disease-related genes in OSCC.

Locating at 15qn5, PDIA3 is reported as a protein disulfide isomerase (PDI). It can modify and fold newly synthesized glycoproteins by forming disulfide bonds between certain residues within these proteins[37]. Recent studies have shown that PDIA3 regulates cell invasiveness in cervical cancer and that a high level of PDIA3 is associated with a low patient survival rate. As such, PDIA3 was even proposed as a prognostic marker for cervical cancer[38]. Similar effects of PDIA3 in gallbladder carcinoma have been reported by Qiong Zuo, et al.[39]. Furthermore, PDIA3 has also been reported to be differentially expressed in various cancers, including breast carcinoma and ovarian cancer[40,41]. In this study, no PDIA3 expression was detected in normal oral mucosa, while a high percentage of positive expression and strong staining was found in OSCC. There are four DEGs directly connected to PDIA3 and enriched in the focal adhesion and ECM-receptor interaction pathways. Previously, PDIA3 was demonstrated to interact with specific DNA sequences in both HeLa cells and a melanoma cell line[42]. Thus, the slight up-regulation of PDIA3 may induce the significant up-regulation of a group of target genes.

Being produced by normal alveolar macrophages and granulocytes, Matrix metalloproteinase 9 (MMP9) plays an essential role in local proteolysis of the extracellular matrix (especially collagens IV and V) and in leukocyte migration[43]. There is strong clinical and experimental evidence of an association between elevated levels of matrix metalloproteinase MMP9 and cancer progression, metastasis and shortened patient survival, as it plays a key role in tumor cell migration by digesting the basement membrane and ECM components[39]. MMP9 has also been reported to accelerate tumor growth by facilitating angiogenesis in the tumor[44] and is thought to promote tumor invasion in HNSCC cell lines[45]. Some studies have also indicated that proteolytic activities are elevated in OSCC compared to normal oral mucosa[46]. An investigation of the relationship between clinical characteristics and MMP9 expression in laryngeal squamous cell carcinoma found that MMP9 expression has found to be correlated with high histopathology grade, stage, metastatic potential, recurrence potential, and low survival[47]. In our analysis, MMP9 displays an important node in the network and its expression progressively increased from NOM to OLK to OSCC.

BGH3, transforming growth factor-beta-induced protein (also known as TGFBI), is a secretory protein whose expression is induced by TGF-β in a variety of cell types. It contains 4 fasciclin-1 (FAS1) domains and a carboxy-terminal Arg-Gly-Asp (RGD) motif, which binds to type I, II and IV collagens, fibronectin and integrin. BHG3 also plays a key role in cell-collagen interactions. The effect of BGH3 on tumor progression is still controversial, but it has been suggested to act as either a tumor suppressor or an oncogene depending on the origin of the cancer. BGH3 has been reported to be down-regulated in breast, ovarian, and lung cancer but over-expressed in clear cell renal carcinoma, pancreatic cancer, colorectal cancer and OSCC[48]. Divya Bhagirath et al. reported that BGH3 is over-expressed in the serum and urine of urothelial carcinoma patients[49]. However, the mechanism of the BGH3-tumor interaction remains unclear. Some reports have shown that BGH3 inhibits tumor cell migration by interacting with other ECM components and regulating integrin expression and that high expression of BGH3I increases the sensitivity of tumors to chemotherapy[50]. In contrast, other reports have found that BGH3 is responsible for tumorigenesis and promotes tumor metastasis by stimulating tumor cell adhesion[51]. A recent study of myeloma showed that hypermethylation of the BGH3 gene was associated with tumor promotion[52]. In our study, BGH3 increased sharply from normal oral mucosa to precancerous lesions to carcinoma.

Our correlation analysis suggests a positive linear association between MMP9 and BGH3 expression in OSCC and pre-OSCC samples (Table 3). Yeon Hyang Kim et al. found that BGH3 was cleaved by MMP-9, and its cleavage resulted in changes in its binding properties and cell adhesion, cell migration, FAK/Src signal, and chemoattractant effects[53]. The authors finally concluded that MMP9-cleaved BGH3 plays a crucial role in MMP9-mediated tumor migration. To the best of our knowledge, we are the first to report a correlation between MMP9 and BGH3 at the protein level in OSCC.

In summary, we explored and confirmed the consistent up-expression of BGH3, MMP9 and PDIA3 in OSCC development based on a large-scale bioinformatics analysis and solid validation. Coupled with future validation, the 3 genes might be further developed into potential biomarkers for early diagnosis of OSCC disease. On top of the traditional DEGs, investigating the hub genes interacting with the majority of the DEGs often provides interesting aspects of disease development. Usually the number of collected samples is frequently limited for each individual research group, but here we show that integrative analysis of large sample sets and validation on different experimental platforms might be feasible owning to fast accumulation of on-line transcriptomics database such as GEO or ArrayExpress. At last, our analysis methods and workflow can be easily applied to other types of disease to identify potential biomarkers.

## Supporting Information

S1 Table Characteristics of the 6 selected microarray datasets. (DOCX)

S2 Table Clinical parameters of patients and protein positive expression rates for NOM, OLK and OSCC. (DOCX)

S1 File Information of DEGs. There are three supplementary sheets. Table A, information about the common DEGs in group I and group II. Table B, information about the DEGs in group I. Table C, information about the DEGs in group II.
(XLSX)

S2 File Information of modules and hub genes in ppi network. Table A, information about three primary modules. Table B, parameters of nodes in module B.
(XLSX)
