# Supplementary material for: Largescale Transcriptomics Analysis Suggests Over-Expression of BGH3, MMP9 and PDIA3 in Oral Squamous Cell Carcinoma
Source: PLoS One. 2016 Jan 8;11(1):e0146530. doi: 10.1371/journal.pone.0146530 (PMC4706424; doi:10.1371/journal.pone.0146530)
Supplement: S1 Table — (DOCX) [file pone.0146530.s001.docx]

**Table S1. Characteristics of the 6 selected microarray datasets.**

| Chip Data  (group type) | Data Type | Sample Size | Array Platform |
| --- | --- | --- | --- |
| **GSE9844**  **(I)** | Oral tongue squamous cell carcinoma | 26 OTSCC samples,12 control samples | Human Genome U133A Plus 2.0 |
| **GSE30784**  **(I)** | oral squamous cell carcinoma | 167 OSCC samples,45 control samples | Human Genome U133A Plus 2.0 |
| **GSE31056**  **(I)** | oral tongue squamous cell carcinoma | 23 tumor samples,73 control samples | Human Genome U133A Plus 2.0 |
| **GSE3524**  **(II)** | oral squamous cell carcinoma | 16 OSCC samples,4 control samples | Human Genome U133A |
| **GSE13601**  **(II)** | oral tongue squamous cell carcinoma | 31 OTSCC samples,26 control samples | Human Genome U95Av2 |
| **GSE23036**  **(II)** | head and neck squamous cell carcinoma | 63 HNSCC samples,5 normal samples | Human Genome U133A2.0 |
