# Supplementary material for: Largescale Transcriptomics Analysis Suggests Over-Expression of BGH3, MMP9 and PDIA3 in Oral Squamous Cell Carcinoma
Source: PLoS One. 2016 Jan 8;11(1):e0146530. doi: 10.1371/journal.pone.0146530 (PMC4706424; doi:10.1371/journal.pone.0146530)
Supplement: S2 Table — (DOCX) [file pone.0146530.s002.docx]

**Table S2. Clinical parameters of patients and protein positive expression rates for NOM, OLK and OSCC.**

| Group | n | Male | Female | Age(±sd) | Positive Rate(%) | | |
| --- | --- | --- | --- | --- | --- | --- | --- |
|  |  |  |  |  | MMP9 | BGH3 | PDIA3 |
| NOM | 12 | 5 | 7 | 42.6(±10.6) | 11/12 | 7/12 | 0/12 |
| OLK | 20 | 16 | 4 | 57.9(±9.6) | 16/20 | 17/20 | 14/20 |
| OSCC | 35 | 22 | 13 | 63.1(±13.1) | 31/35 | 30/35 | 35/35 |
